# Riluzole Administration to Rats with Levodopa-Induced Dyskinesia Leads to Loss of DNA Methylation in Neuronal Genes

**DOI:** 10.3390/cells10061442

**Published:** 2021-06-09

**Authors:** Luca Pagliaroli, Abel Fothi, Ester Nespoli, Istvan Liko, Borbala Veto, Piroska Devay, Flora Szeri, Bastian Hengerer, Csaba Barta, Tamas Aranyi

**Affiliations:** 1 Department of Molecular Biology, Semmelweis University, H-1094 Budapest, Hungary; pagliaroli.luca@gmail.com (L.P.); barta.csaba@med.semmelweis-univ.hu (C.B.); 2 Institute of Enzymology, Research Centre for Natural Sciences, 1117 Budapest, Hungary; fothi.abel@ttk.hu (A.F.); istvanliko@gmail.com (I.L.); vetoborbala@gmail.com (B.V.); piroskadevay@mac.com (P.D.); szeri.flora@ttk.hu (F.S.); 3 CNS Department, Boehringer Ingelheim Pharma GmbH & Co. KG, 88400 Biberach an der Riss, Germany; ester.nespoli@uni-ulm.de (E.N.); bastian.hengerer@boehringer-ingelheim.com (B.H.); 4 Department of Child and Adolescent Psychiatry/Psychotherapy, University of Ulm, 89081 Ulm, Germany; 5 Department of Biochemistry, Semmelweis University, 1094 Budapest, Hungary

**Keywords:** dyskinesia, levodopa, Riluzole, abnormal involuntary movements, Tourette syndrome, epigenetics, DNA methylation, Reduced Representation Bisulfite Sequencing (RRBS)

## Abstract

Dyskinesias are characterized by abnormal repetitive involuntary movements due to dysfunctional neuronal activity. Although levodopa-induced dyskinesia, characterized by tic-like abnormal involuntary movements, has no clinical treatment for Parkinson’s disease patients, animal studies indicate that Riluzole, which interferes with glutamatergic neurotransmission, can improve the phenotype. The rat model of Levodopa-Induced Dyskinesia is a unilateral lesion with 6-hydroxydopamine in the medial forebrain bundle, followed by the repeated administration of levodopa. The molecular pathomechanism of Levodopa-Induced Dyskinesia is still not deciphered; however, the implication of epigenetic mechanisms was suggested. In this study, we investigated the striatum for DNA methylation alterations under chronic levodopa treatment with or without co-treatment with Riluzole. Our data show that the lesioned and contralateral striata have nearly identical DNA methylation profiles. Chronic levodopa and levodopa + Riluzole treatments led to DNA methylation loss, particularly outside of promoters, in gene bodies and CpG poor regions. We observed that several genes involved in the Levodopa-Induced Dyskinesia underwent methylation changes. Furthermore, the Riluzole co-treatment, which improved the phenotype, pinpointed specific methylation targets, with a more than 20% methylation difference relative to levodopa treatment alone. These findings indicate potential new druggable targets for Levodopa-Induced Dyskinesia.

## 1. Introduction

Hyperkinetic movement disorders, or dyskinesias, are a variable group of disorders characterized by excessive and abnormal repetitive involuntary movements due to dysfunctional activity of the basal ganglia, cerebral cortex, cerebellum, and other motor pathways. Dyskinesias arise from widely different etiologies, ranging from neurodevelopmental alterations (e.g., tics in Tourette syndrome), genetic abnormalities (e.g., dystonia), neurodegenerative diseases (e.g., chorea in Huntington’s disease), and administration of drugs (e.g., Levodopa-Induced Dyskinesia in Parkinson’s disease) [1]. 

Levodopa-Induced Dyskinesia (LID) develops in almost 80% of patients with Parkinson´s disease (PD) following 5–10 years of levodopa treatment (L-DOPA). Once established, LID becomes a disabling symptom that severely hampers the quality of life [2,3]. There are different strategies to manage the manifestation of LID: administration of lower doses of L-DOPA, treatment with (extended-release) amantadine, and antiepileptic [4,5,6,7,8], anti-psychotic [9], or anti-glutamatergic drugs [10,11]. However, all these treatments show inconsistent results coupled with side effects indicating the need for better solutions. Deep-brain stimulation is a further, but rarely used, strategy due to its invasive nature [12,13]. This makes LID a major focus of research, especially considering that L-DOPA treatment in Parkinson´s disease is unavoidable, as it supplies the striatum, a major component of the basal ganglia involved in motor function, with the dopamine it lacks after the neurodegeneration of the dopaminergic neurons located in the substantia nigra [14].

The pathological mechanism of LID is still not fully understood. Fortunately, LID has been successfully modelled for over a decade in rodents of different ages and primates [15,16]. In the LID model, toxin-induced neurodegeneration of nigrostriatal dopaminergic neurons and the subsequent chronic treatment with L-DOPA induce abnormal involuntary movements (AIMs) [17]. Thus, this model provides a sound basis for the investigation of the molecular mechanisms and testing new therapeutic approaches. It has been shown that the activation of the dopamine D1 and D2 receptor cAMP-PKA pathway [18,19] and the glutamatergic and serotoninergic neurotransmission, ERK signaling, are involved in the pathogenesis of LID [20,21,22,23].

Riluzole, a neuroprotective and glutamate transmission modulator used to treat amyotrophic lateral sclerosis, has proven its efficacy in modulating AIMs in rodent models [24,25,26]. Moreover, Riluzole was also shown to be effective in treating motor disorders seen in Huntington’s disease or multiple system atrophy [27,28] and was tested in PD with controversial effects. Although the mechanism of action of Riluzole is still not fully understood, it mostly exerts anti-glutamatergic functions, but it can also modulate the release of acetylcholine, dopamine, serotonin, and GABA through mechanisms independent of glutamate receptors, and inhibit tetrodotoxin-sensitive Na+ channels [21,29]. One of the most interesting peculiarities of Riluzole in clinics is its safe profile for adolescents as well making it a promising drug for many tic/movements disorders [27].

In recent years, there has been considerable evidence suggesting that hyperkinetic/tic movement disorders may arise not only from the well-defined genetic mechanisms, but also from underlying epigenetic mechanisms [30,31,32,33].

The latter mechanisms regulate gene expression through the covalent modifications of chromatin resulting in the opening or closing of the structure, and hence are considered ‘epi’ genetic as they exert their effect without modifying the DNA sequence. DNA methylation is an extensively studied epigenetic modification with clinical relevance [34]. In mammals, methylation occurs primarily in CpG dinucleotides [35], enriched in regions of the genome with short CG-rich sequences (CpG islands, CGI) frequently found in regulatory sequences of genes [36,37]. Therefore, in addition to repeat elements of the genome, DNA methylation targets primarily promoters and enhancers and regulates gene expression. Indeed, methylation of these regions predominantly inhibits the transcription of genes. Although DNA methylation is essential for the maintenance of long-term neuronal and behavioral memory, the methylation pattern can change as a consequence of environmental stimuli leading to tissue-specific methylation [38,39]. As DNA methylation can dynamically change under various conditions [39,40,41,42], it was suggested to play a major role in the development of LID [33].

Although it has been suggested and shown that LID can influence DNA methylation, very few studies are available to date, and no effect on methylation of the signal transduction pathways was observed. Therefore, in our study, we investigated the changes in DNA methylation that might play a role in the appearance of AIMs in a LID model. The effect of Riluzole on DNA methylation has never been studied before. Hence, we also studied the rat model treated with Riluzole to identify potential new targets for LID.

## 2. Materials and Methods

### 2.1. Abnormal Involuntary Movements (AIMs) Rat Model

**Figure 1:**
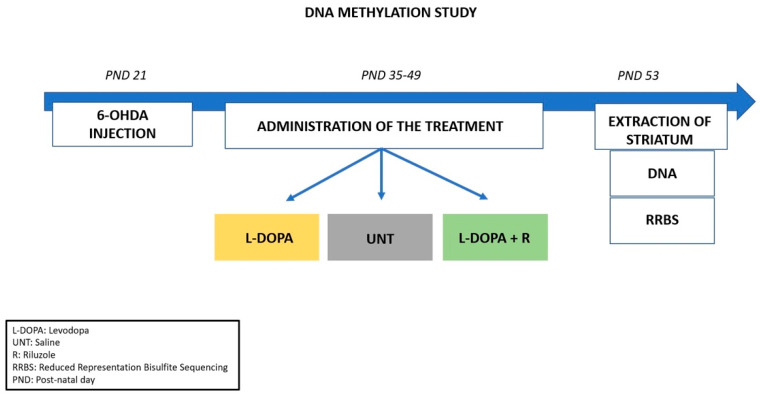
Schematic representation of the experimental workflow. Rats received a unilateral injection of 6-OHDA at PND 21 followed by a 2-weeks sub-chronic treatment (from PND 35 to PND 49) according to their respective group (L-DOPA, UNT, L-DOPA + Riluzole). The rats received one final treatment at PND 53; striata were collected and analyzed for changes in methylation levels.

In this study, we used samples from an Abnormal Involuntary Movements (AIMs) animal model described in our previous paper [16]. As shown in Figure 1, juvenile male Wistar rats underwent a unilateral stereotaxic surgery with the injection of 6-Hydroxydopamin (6-OHDA) into the left medial forebrain bundle at post-natal day (PND) 21. Two weeks after surgery (PND 35), the animals were randomly divided into 3 groups and received a chronic treatment (6 times in 2 weeks, intra-peritoneally (i.p.)). The first group was administered with saline (UNT); the second group (L-DOPA) was chronically administered with a solution of L-DOPA methyl ester hydrochloride and benserazide (Sigma-Aldrich Chemie GmbH, Taufkirchen, Germany) in saline (6/15 mg/kg, i.p.), while the third group (L-DOPA + R) was administered L-DOPA/benserazide (6/15 mg/kg, i.p.) and Riluzole (Sigma-Aldrich Chemie GmbH, Germany) dissolved in 1% Tween (6 mg/kg, i.p.). At the end of the chronic treatment at PND 53, the animals were sacrificed two hours after the administration of a final pharmacological treatment. The brain was removed and contralateral (control) and ipsilateral (lesioned) striata were extracted, snapped frozen in liquid nitrogen, and stored at −80 °C.

### 2.2. AIMs Scoring

AIMs scoring followed a well-established rating scale [43] according to which AIMs are subdivided into 3 subtypes: limb dyskinesia, axial dystonia, and orolingual dyskinesia. Each subtype was scored on a scale from 0 to 4 (0 = absent; 1 = occasional; 2 = frequent; 3 = continuous but interrupted by sensory distraction; 4 = continuous and severe, not interrupted by sensory distraction). As described in our previous paper [16], subsequent to the administration of the drug treatment, the rats were individually observed for 1 min every 20 min for 180 min to detect the presence of AIMs. The AIM score was generated as the sum of the independent scores of each body part per time point.

### 2.3. Reduced Representation Bisulfite Sequencing (RRBS)

Three rats per group were randomly chosen for the analysis. Recently published RRBS studies often use a similar or smaller number of animals for one or several/all groups [44,45,46,47,48,49,50,51].

Striata of the lesioned and contralateral sides were investigated by reduced representation bisulfite sequencing (RRBS) [52]. Genomic DNA was extracted using the AllPrep DNA/RNA/miRNA Universal Kit (QIAGEN) following manufacturer’s instructions. A premium RRBS kit (diagenode.com) was used for RRBS library preparation [53]. Briefly, 100 ng DNA was digested with MspI restriction enzyme which cuts DNA at C^CGG sequences regardless of the DNA methylation status. After adaptor ligation and size-selection, up to 6 samples were pooled according to the manufacturer’s instructions. Pooled samples were bisulfite treated, purified, and amplified resulting in high coverage for CpG islands and promoter regions while also retaining significant coverage of other genomic elements. The final libraries were quantified using the Qubit dsDNA HS Assay (Life Technologies), and the library profile was checked on the Bioanalyzer 2100 (Agilent). Libraries were then sequenced on the Illumina HiSeq 2000 platform (Single end 50 bp).

### 2.4. Bioinformatic Analysis

Illumina sequencing reads were quality checked with the FastQC (v0.10.0) software. The reads were adapter trimmed by trimgalore (v0.4.0), and an additional two nucleotides were removed from their 3′ ends using the *–rrbs* option. Trimmed reads were mapped to the rn6 genome using Bismark (v0.14.3.) tolerating one non-bisulfite mismatch per read (-n 1 option). After mapping, sam files were sorted by samtools (v1.4.1) and used by the MethylKit (v1.8.0) for further analysis. First, methylated and unmethylated Cs in CpG context were read in. Cs with 9< reads were kept. Similarity of samples was checked by calculating pairwise Pearson’s correlation. Differentially methylated sites (DMS) were extracted by using the calculateDiffMeth function with an overdispersion correction and the Chi square test without a threshold for a minimum difference, but with q < 0.01 significance [54]. DMSs were annotated according to the type of genomic region where they are located (promoter, exon, intron, intergenic or CpGisland, CpGshore, other). Hypo- and hypermethylated chromosomal positions were further annotated to obtain the nearest ENTREZ and RefseqIDs, Gene Names. DMS density was analyzed with 1 MB sized genomic bins.

## 3. Results

### 3.1. DNA Methylation Analysis

As described in our previous report [16], the chronic administration of L-DOPA after a unilateral 6-OHDA lesion induced strong abnormal involuntary movements in juvenile rats. The phenotype was partially rescued by the administration of Riluzole. Here, we used tissue samples from the striata of the rats of the previous study to uncover the role of DNA methylation in the development of the phenotype. We performed reduced representation bisulfite sequencing (RRBS), which is a high-throughput technique for detecting methylation at a single nucleotide resolution of the most CpG-rich regions of the genome.

**Figure 2:**
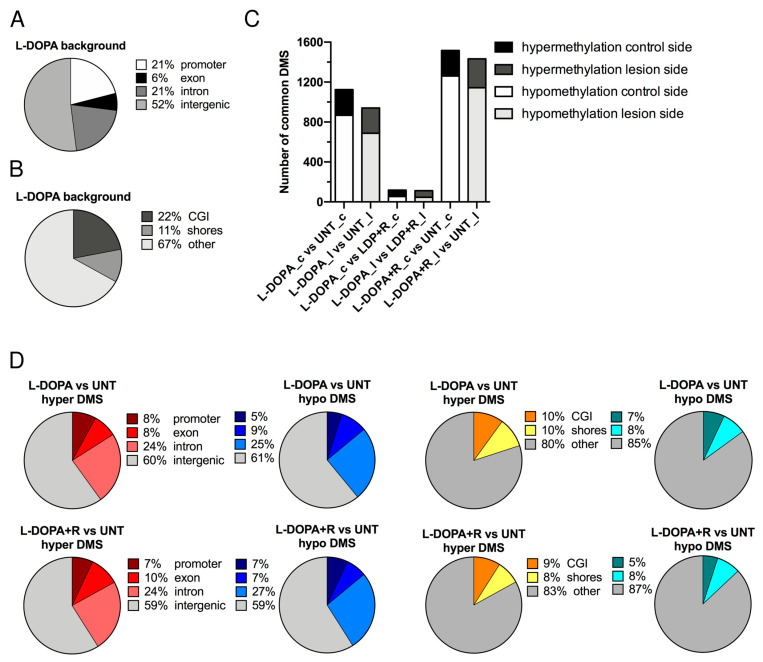
Distribution of CpGs detected by RRBS in the rat genome. (**A**) Distribution of CpGs in the genome (promoter, exon, intron, intergenic) (**B**) Distribution of CpGs in areas of different CG density (CpG island, CpG shores, other). (**C**) Number of differentially methylated sites (DMSs) in the experimental groups (L-DOPA vs. UNT control side, L-DOPA vs. UNT lesioned side, L-DOPA vs. L-DOPA + R control side, L-DOPA vs. L-DOPA + R lesioned side, L-DOPA + R vs. UNT control side, and L-DOPA + R vs. UNT lesioned side) classified as hyper- or hypomethylated. (**D**) Genomic distribution of CpG methylation changes. Distribution of hypermethylated and hypomethylated CpGs in L-DOPA treated vs. UNT rats. Distribution of hypermethylated and hypomethylated CpGs in L-DOPA + Riluzole treated vs. UNT rats.

First, we compared the lesioned and the control sides of striata of three rats, which underwent only 6-OHDA lesion (untreated, UNT). The control for the success of the surgery and the unilateral toxin injection was the contralateral striatum. A set of approximately 435,000 CpGs was present in all six striata (three animals, both lesion and control sides). Figure 2A shows the distribution of these 435,000 CpGs in the rat genome. Half of them were located in intergenic regions. In promoters and introns, we found an approximately equal amount (~20% each). In the L-DOPA and L-DOPA + Riluzole (L-DOPA + R) groups, the genome-wide distribution of CpGs identified in all six striata by RRBS was similar. Figure 2B also shows the genome-wide distribution of the 435,000 background CpGs relative to the genomic GC density. The most CpG-dense regions are CGIs. CpG shores have generally intermediate CpG density and are located within 2 kb of CGIs. The majority of the investigated CpGs (>60%) were located outside CpG islands and CpG shores.

#### 3.1.1. Differentially Methylated Sites (DMS)

To identify the effect of the 6-OHDA injection alone or in combination with L-DOPA or L-DOPA + R administration on striatal DNA methylation, we compared the methylation of the three lesioned to the three control sides within each group and identified the differentially methylated sites (DMS). DMS were designated as CpG dinucleotides with a significant methylation-level difference after correction for multiple testing (q < 0.01) (Supplementary Table S1a). Altogether we detected infinitesimal changes, since less than 0.02% of all investigated CpGs were identified as DMS regardless the type of treatment administered. We concluded from these results that the two striata of an animal have almost identical DNA methylation profiles. Furthermore, we observed that the various treatments have no differential effect on the DNA methylation pattern of the lesioned and control striata.

Since the lesion alone had no effect either on the behavior of the animals or on the methylation of their striata, we decided to compare the DNA methylation between the different groups. We compared first the control striata from the three animals of the L-DOPA treated group with the control striata of the untreated (UNT) animals. As indicated in Supplementary Table S1b, we detected 1125 DMS. Interestingly, more than three times more hypo- than hypermethylated DMS were observed. Very similar results were obtained when we compared the striata of the lesioned sides of the same animals. When we compared the control striata of the L-DOPA + R with the controls of UNT rats, we identified even more DMS. Furthermore, the number of hypermethylated DMS remained almost unchanged, while the hypomethylated DMS increased significantly. Again, very similar results were observed when the lesioned sides were compared (Figure 2C).

Surprisingly, when the L-DOPA and the L-DOPA + R groups were compared, very few DMS were detected, only slightly superior in number to the comparisons described in the Supplementary Table S1a. No major differences were seen between the number of hypomethylated and hypermethylated DMS and the distribution between the lesion and control sides (Supplementary Table S1b). Altogether these data further confirm that the two striata of an animal have almost identical methylation profiles. Furthermore, they also strengthen our observation that the 6-OHDA lesion induces only very few DNA methylation changes, while the systemic drug treatments have a similar effect on both striata of an animal irrespective of the previous surgery. In order to further prove this, we matched the DMS of the control and lesioned sides in the comparisons of the different groups of animals. As expected, this comparison showed that the DMS of either the control or the lesioned side behave very similarly and have almost identical methylation patterns (Figure 2C). 

#### 3.1.2. Genomic Distribution of DMS

Based on the high similarity of the methylation levels of the individual CpGs of the two striata in the same animal, we decided to combine the three lesioned and three control sides of the animals undergoing the same treatment in one group. In the next step, we compared the methylation profile of the six L-DOPA and the six UNT striata. In order to increase the number of CpGs investigated during the analysis, we relaxed our criteria. Indeed, for the inclusion of each CpG, we required useful sequence information of at least four out of the six striata from each group analyzed. In this way, while we maintained the stringent analyses criteria [54], we could substantially increase the number of CpGs to more than 600,000 without altering the genome-wide distributions of this new background.

We observed a globally increased number of DMS compared to the previous analyses when lesioned and control sides were separately compared. The overall increased number of hypomethylated DMS was still predominant (Supplementary Table S1c). When we carried out genome-wide distribution analyses, we observed that DMS are somewhat enriched in gene bodies and intergenic regions, while we identified a much more important absence of DMS in gene promoters (Figure 2D). A similar non-random distribution of DMS characterized CGIs, shores, and “other”, also known as “open-sea” regions. The predominant portion (>80%) of DMS were located in “open-sea”, i.e., CpG-poor regions, showing a big increase relative to the background distribution. Furthermore, almost three times fewer DMS were identified in CGIs than one would expect based on random distribution (Figure 2D).

Next, we performed the comparison of the six L-DOPA + R and UNT striata. Again, similar results were observed to that detected in the previous analysis. The genome-wide distributions of DMS were strikingly similar to those identified in the L-DOPA versus UNT groups (Figure 2D). When we compared the L-DOPA and L-DOPA + R animals, we observed only a few DMS. Furthermore, only slight skewing was observed toward hypo- or hypermethylation (179 vs. 118 DMS) with a still reduced representation of promoters.

**Figure 3:**
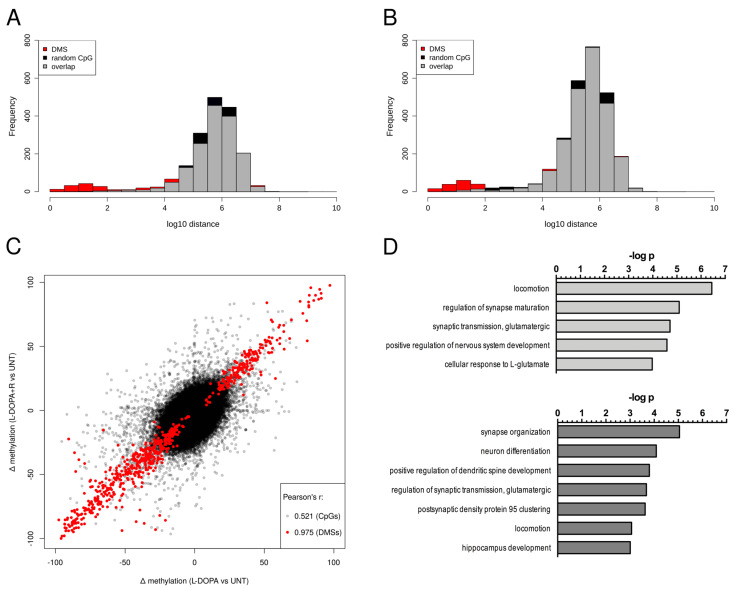
Characterization of DMS. (**A**,**B**) Distribution of genomic distances between neighboring DMS in L-DOPA vs. UNT and L-DOPA + R vs. UNT groups shown in red. The average of 100 random distributions of genomic distances of the same number of CpGs is shown in black. The overlap is shown in grey. The small difference in the shape of the random distributions between the two panels is due to the higher number of DMS in the L-DOPA + R vs. UNT comparison leading to shorter average distances between two random CpGs. (**C**) Scatter plot of CpGs present in both L-DOPA vs. UNT and L-DOPA + R vs. UNT comparisons. The plot shows the methylation changes in the treated animals of each CpG relative to the UNT animals. The red dots indicate CpGs, which are DMS in both comparisons. Pearson’s R values are also shown in grey and red. (**D**) shows the results of the GO term enriched analysis performed using DMS detected in L-DOPA vs. UNT (**upper panel**) and L-DOPA + R vs. UNT (**lower panel**) comparisons.

We also investigated the physical genomic distances of DMS identified in the L-DOPA and in the L-DOPA + R UNT comparisons relative to a random distance distribution (obtained by 100 runs of random sampling) (Figure 3A,B). Our results clearly showed that both in L-DOPA and L-DOPA + R groups, there was an enrichment of DMS relative to a random distribution within 100 bp distance, i.e., CpGs in close proximity tend to undergo significant methylation changes suggesting most probably the co-occurrence of similar changes. Indeed, >85% of DMS within 100 bp distance underwent methylation change in the same direction. Furthermore, we detected a very similar amplitude of methylation change in most of the DMS in close proximity.

In the next step, we carried out a genome-wide analysis and generated scatter plots to compare the methylation level differences of L-DOPA vs. UNT and L-DOPA + R vs. UNT. As a result of this analysis, we were able to observe a clear correlation between the methylation level of CpGs in the treated animals (r = 0.52) (Figure 3C). Furthermore, when we restricted our analyses to DMS in at least one of the drug-treated groups relative to UNT rats, we observed a striking correlation of DNA methylation between the drug-treated groups of animals (r = 0.89). This methylation correlation increased to r = 0.98 when we considered only CpGs being DMS in both drug-treated groups relative to UNT animals (Figure 3C). Altogether these data further suggested that the methylation changes are primarily due to the repeated L-DOPA treatment and stay unaltered by the end of the treatment.

#### 3.1.3. Genes Undergoing DNA Methylation Alteration in the LID Model

Next, we carried out a systematic annotation of the DMS to genes (Supplementary Table S1d,e). As already mentioned, due to the relatively small number of DNA methylation changes in the promoters, most of the identified gene-related DMS were located in gene bodies. To identify the role of the genes with DMS, we performed a gene enrichment analysis by using the Gene Ontology enRIchment anaLysis and visuaLizAtion (GOrilla) tool [55] (Figure 3D). In these analyses, we compared the genes with at least one DMS (either hypo or hyper) to the background gene set we analyzed: all the genes with at least one CpG were included in the comparison. This background contained approximately 13,500 genes, 12,963 of which were associated with GO terms. The L-DOPA vs. UNT comparison identified 554 genes associated with both DMS and GO terms. After the analysis, several significantly enriched GO terms were directly related to neurodevelopment (particularly synapse-related terms) or glutamatergic neurotransmission. Examples include regulation of synapse maturation or organization (e.g., the following genes: *Camk2b*, *Neurexin 1*, *Reln*), the glutamate receptor signaling pathway (several glutamatergic/both metabotropic and ionotropic/receptor coding genes: *Grm4*, *Grm8*, *Grik3*, *Grik4*, etc.), neuron remodeling (*Netrin 4*, etc.), and locomotion. Multiple-enriched GO terms were general ones, which could also be related to neurodevelopment and neuronal signaling (e.g., cell adhesion, developmental process, cell projection assembly, etc.).

Similar results were obtained when the 834 genes with DMS and GO terms from the L-DOPA + R comparison were investigated (Figure 3D). The background being almost identical, approximately the same number of GO terms could be studied (12,900). Again, several of the significantly enriched GO were directly related to “synapse” (e.g., synapse organization (examples: *Neuregulin*, *Synaptopodin*, *Neurexin 1* and *2*), neuron differentiation (*Cacna1a* and genes involved in epigenetic modifications (DNA methyltransferases 3a—*Dnmt3a*) and others), chemoattraction of axon (*Robo3*), dendrite development or glutamatergic neurotransmission (regulation of AMPA receptor activity (*Cacng4*, *Cacng8*), etc.). Interestingly, locomotion as the GO term, which was the second most significantly enriched in the L-DOPA group, was much less represented in the L-DOPA + R gene set.

#### 3.1.4. Identification of DMS with Differential Methylation between L-DOPA and L-DOPA + Riluzole Treated Animals

Although as mentioned above the L-DOPA and L-DOPA + R treatments resulted in very similar DNA methylation patterns, the rats exhibited different phenotypes [16]. Therefore, we investigated the potential methylation changes, which could account for the observed differences. As mentioned earlier, more DMS were detected in the L-DOPA + R than in the L-DOPA group. When we compared the L-DOPA and L-DOPA + R animals, we observed only a few DMS with a slight preference for hypomethylation (179 vs. 118 DMS) still with a reduced representation of promoters. These 297 DMS were located in 98 genes, 12% of which are linked to PD or the dopaminergic system according to literature data (Supplementary Table S1f).

**Figure 4:**
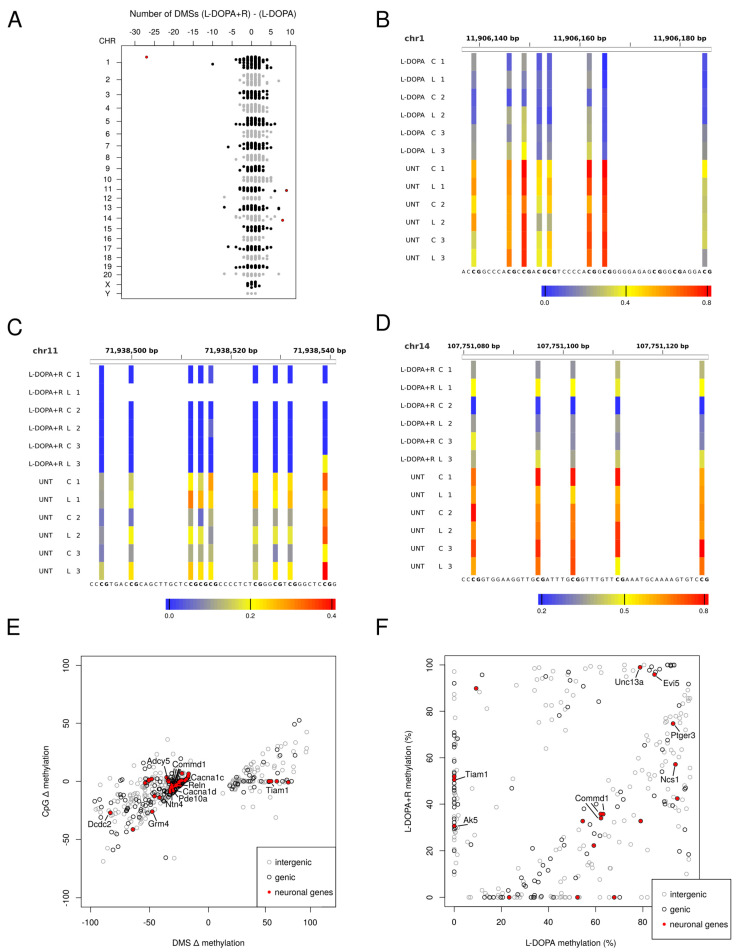
Comparison of DMS from L-DOPA vs. UNT and L-DOPA + R vs. UNT. (**A**) Difference of the number of DMS/bin of 1 Mb between L-DOPA + R vs. UNT and L-DOPA vs. UNT comparisons. Each dot corresponds to one bin. Black and grey colors alternate throughout the different chromosomes, numbered on the left side of the panel. (**B**–**D**) Methylation of selected genomic regions from bins indicated by red dots on panel (**A**) (**B**: Chr1: 5S rRNA, **C**: Chr11: *Pigx*, and **D**: Chr14: *Commd1* genes) (**E**) Plot of methylation changes are shown for DMS from either L-DOPA + R vs. UNT or L-DOPA vs. UNT comparisons. Only DMS from CpGs analyzed in both comparisons are shown if the methylation difference between L-DOPA and L-DOPA + R samples is at least 20%. Red dots indicate DMS of genes with relevance to LID. Grey circles indicate intergenic DMS. (**F**) DMS of L-DOPA vs. L-DOPA + R comparison. DMS of genes with relevance are indicated in red.

When investigating the differences further, we realized that the density of DMS was different in some genomic regions between the two comparisons (L-DOPA vs. UNT and L-DOPA + R vs. UNT, although only a few L-DOPA vs. L-DOPA + R DMS were identified). To study these densities, we created bins of 1 Mb and computed the number of DMS in each bin both from the L-DOPA + R and the L-DOPA comparisons. After subtraction of these counts, which typically gave zero or one as a result as expected due to the similarity of the L-DOPA and L-DOPA + R groups, we determined the genomic regions, which showed the highest number of DMS differences between the two treatments (Figure 4A).

The most impressive 1 Mb genomic region (>25 DMS number difference) is located on chromosome 1 in the region close to the 5S rRNA gene in the L-DOPA samples (Figure 4A,B). However, more DMS were detected with than without Riluzole treatment, leading to bins with more DMS in the combined treatment group. For example, such a region is the *Pigx* gene, containing 9 DMS in the combined and none with the single treatments (Figure 4C). Similarly, the Commd1/Zrsr1 imprinted region interestingly undergoes DNA methylation alteration upon the L-DOPA + R treatment (Figure 4D).

In addition to the DMS density variability, some absolute DNA methylation differences could also be identified, and additional ones suspected. Therefore, we looked for CpGs, which underwent significant changes (DMS) in either the L-DOPA or L-DOPA + R comparison with UNT and where the absolute methylation change difference between L-DOPA and L-DOPA + R was equal to or higher than 20%. A total of 497 such CpGs were identified (Figure 4E,F). From these DMS, altogether 180 were found in 171 genes. After a detailed search of the literature, more than 20% of the genes in this set show strong evidence for having an important role in neurodevelopmental processes or neuropsychiatric disorders. However, the majority of these genes are not directly related to glutamatergic or GABAergic neurotransmission or tetrodotoxin-sensitive Na+ channels, which are directly linked to the known molecular action of Riluzole, suggesting that further direct or indirect targets of the drug exist in the striatum.

In summary, our data show that chronic L-DOPA treatment in juvenile rats upon a 6-OHDA lesion leads to DNA methylation alterations in the striatum. Furthermore, the L-DOPA + R treatment improved the phenotype induced by L-DOPA, and our methylation analysis reveals potential new druggable targets for LID and/or neurodevelopmental diseases.

## 4. Discussion

Levodopa-Induced Dyskinesia (LID) is a generally occurring side-effect of L-DOPA treatment of PD [3]. In the recently developed juvenile rat model, the animals had typical LID (ascertained by the AIM scores) demonstrating a similar phenotype in juvenile and adult rats [16]. As expected, we found that co-administration of Riluzole significantly improved the phenotype. Here we investigated DNA methylation alterations in the tissue samples of the rat striata recently analyzed for transcriptional activity [16].

We used a single-nucleotide resolution approach to study genome-wide DNA methylation. RRBS [53] investigates CG-rich regions and leaves out from the analysis the majority of the genome where the CG dinucleotides are far from each other. RRBS targets regions identified by two MspI restriction enzyme recognition sites (CCGG) in close proximity (200 bp–1.2 kb), which characterizes the CpG-rich regions. We found that fragments as small as 50 bp could be detected, which confers a small bias toward highly CpG-rich regions. We have also sequenced the ends of fragments larger than 1.2 kb; however, these sequences are not expected to contain several CpGs. RRBS investigates only the two 50 bp ends of each fragment without data from the CpGs between the ends, but similar methylation most probably would be detected within the entire region, as suggested by the literature [52]. Our data confirmed this: over 85% of DMS in close proximity (closer than 100 bp) underwent methylation change in the same direction and very often had an almost identical amplitude.

A limitation of our study is the small number of animals investigated per group. In order to circumvent this problem, we used very stringent DNA methylation analyses criteria (overdispersion correction and Chi square test and correction for false discovery rate [54]) to avoid the identification of false positive DMS, which on the other hand decreased the sensitivity and led to the identification of relatively few DMS. Indeed, there were almost no DMS when we compared the two striata of the animals from the same group. Thus, we concluded that the two sides in these animals have only slightly different methylation profiles, which cannot be distinguished by the stringent method we used. When we performed the much less stringent method used in a previous study on old rats, we obtained in our samples approximately 4400 highly dynamic regions (HDR) according to the nomenclature used in that study [32]. This number is almost identical to the number of DMS the authors reported [32].

By using the stringent DMS detection method, we also concluded that the almost identical methylation profiles of the two striata of an animal are not altered by the 6-OHDA lesion affecting only one side. Furthermore, even the systemic L-DOPA or L-DOPA + R treatment had a similar effect on the two striata of the same animal. These are important findings since only sparse data exist on DNA methylation of symmetrical organs in mammals, particularly in the brain. In our case, most probably the very stringent criteria of the DNA methylation analysis and the small number of samples prevented us to detect more side-specific methylation differences.

Our main finding was that chronic L-DOPA treatment led to abundant DNA methylation changes, mostly hypomethylation outside of promoters and CGIs. This methylation pattern alteration has already been reported earlier in rat brain and arteries upon environmental stress [56,57]. We concluded that the chronic L-DOPA treatment was the main cause of DNA methylation alterations, strengthening the hypothesis that LID is associated with methylation changes, which might play a substantial role in the pathomechanism. Surprisingly, the L-DOPA + R had a very similar effect to the L-DOPA treatment on DNA methylation, even if some differences were detected, and more DMS were identified. These data suggest that Riluzole only slightly interferes with the DNA methylation profile and does not result in an intermediate methylation landscape between the L-DOPA and UNT animals. Nevertheless, some genomic regions had much more DMS in the L-DOPA + R group. One of them, the *Pigx* gene, harbors a sequence variant associated with Parkinson’s disease [58]. The gene has a yet unclear physiological function, but the encoded protein is involved in the biosynthesis of glycolipids. The *Commd1* gene regulates copper homeostasis, which was also shown to influence the development of neuropsychiatric diseases and PD [59]. Indeed, COMMD1 encodes a scaffold protein involved in the aggregation of misfolded proteins typically observed in Parkinson’s disease [59,60].

The detailed transcriptional landscape characterization of the juvenile rat striatum revealed the primordial role of the NMDA glutamate receptor-PKC/ERK1/2-CREB axis in the development of LID. Indeed, the alteration of the expression of several CREB target genes, activated in the L-DOPA and reduced in the L-DOPA + R groups, was detected in the striata [16]. Due to our stringent analysis, we could more precisely reveal the methylation alterations than what was reported previously. The GO term enrichment analysis led to the identification of a great number of specific neuronal plasticity, development, and glutamatergic genes undergoing DNA methylation changes. When we investigated the DNA methylation alteration at the single gene level, we realized that there were very few overlaps with the genes identified as transcriptionally altered in our AIM/LID model [16]. Among them, NR4a1 (nurr77/ngfIb) should be mentioned, which is tightly linked to the dopaminergic system and is also involved in the regulation of ERK phosphorylation [61,62]. NR4a1 knockout mice were characterized by increased locomotor activity and higher sensitivity to dopamine and stronger rotational behavior induced by L-DOPA [63].

Though we found only very few overlaps between genes with expression and methylation changes, we identified modified DNA methylation of several genes with a complementary function to those with altered expression. Accordingly, we observed altered methylation of various adenylate cyclase genes (*Adcy5*, *Ac7*, and *Ac10*). *Adcy5* is of particular interest since it is the causative gene of a dyskinesia due to the dominant gain of function mutations [64,65]. *Adcy5* has a high expression level in the dorsal striatum and is also involved in dopaminergic (D1 and D2) signalization of the medial spiny neurons (MSN) of the striatum [65,66]. *Adcy5* was one of the genes in our study, which showed more than a 20% methylation difference between L-DOPA and L-DOPA + R treatments, further suggesting its important role. Additional genes related to the cAMP pathway (*Ak5*, *Ptger3*, *Evi*) also underwent different methylation changes in the L-DOPA and L-DOPA + R groups. While AC5 is responsible for the synthesis, PDE10A degrades cAMP, and the interplay of these enzymes determines its level in the striatal neurons. Indeed, PDE10A, which is considered as an early marker of PD and known to influence locomotor movements as well as the expression of CREB, is strongly expressed in the same cells and observed to be involved in LID [67,68,69]. In our experiments, PDE10A also showed high degree of difference in the methylation level between L-DOPA and L-DOPA + R treatments.

As mentioned above, the ERK pathway is involved in the molecular development of AIM/LID. We revealed altered DNA methylation of NCS1, which plays an important role in the signal transduction between NMDA receptors and ERK [70]. We observed an important methylation difference of NCS1 between the L-DOPA and L-DOPA + R group. Similarly, Tiam1, which plays a crucial role in NMDA-dependent dendritic arborization, also undergoes DNA methylation changes, and again an important methylation difference at this gene between the L-DOPA and L-DOPA + R groups was found.

Several further genes involved in glutamatergic signalization showed methylation changes in our model, sometimes >20% between the L-DOPA and L-DOPA + R groups. We identified DMS in GRIN2D, a member of the NMDA receptor genes, GRIK3 and GRIK4, encoding kainite receptors, and GRM4 and GRM8, encoding metabotropic receptors. The involvement of glutamate neurotransmission was strengthened by DMS in the TARP gene family (CACNG4 and CACNG8), encoding regulators of AMPA receptor activity and Unc13a, involved in glutamatergic synaptic vesicle maturation.

Additional genes involved in neuronal plasticity or neuropsychiatric diseases were also identified as undergoing DNA methylation changes due to L-DOPA treatment (*Camk2b*, *Neurexins* (*1* and *2*), *Reln*, *Dab1*, *Netrin 4*, *Neuregulin*, *Synaptopodin*, and *2*, *Cacna1a*,*b*,*c*,*d genes*, *Robo3*, and *DCDC2*). While some of these genes were previously shown to be linked to Parkinson’s disease (e.g., *Cacna1* genes), most of them were not yet reported to play a role in LID or PD. The *Cacna1* genes encode Ca2+ channels, and we observed DMS with a >20% methylation difference between L-DOPA and L-DOPA + R rats. Reelin, associated with neuropsychiatric and neurodegenerative disorders and encoded by the *Reln* gene, influences neuronal migration in the developing brain and regulates AMPA receptor activity [71,72,73]. *Dab1*, an interactor partner of Reelin, which was suggested previously to play a role in LID, underwent similar methylation changes [32]. Neurexin 1 (*NRXN1*), which encodes a pre-synaptic cell-adhesion molecule, is involved in the growth of synapses and in the transmission of glutamate and GABA signals. The relevance of the genes identified in our study is also underlined by a previous report describing the methylation alterations observed in a large cohort of patients with tic-disorder [74]. The nigrostriatal axis also plays an important role in the pathomechanism of tic-disorders and Tourette syndrome. Surprisingly, from the top 12 genes identified by this array hybridization approach, five overlapped with genes detected in our present study. Additionally, both Neurexin 1 and Netrin 4 have also been implicated in the etiology of Tourette syndrome [75,76]. Altogether we consider that the sensitive RRBS method we used in this study was able to identify genes and pathways involved in LID and in the effect of Riluzole (e.g., glutamate neurotransmission, the PKA/ERK pathway, and genes involved in neuronal plasticity). Some of these pathways were already known to play a role in LID, but some specific genes were not yet reported and were not observed as differentially expressed. The most probable explanation for this is their small but relevant expression changes, which should be investigated by more sensitive methods.

## 5. Conclusions

Although the general methylation profile of the L-DOPA and L-DOPA + R groups were very similar to each other, some of the genes harbored DMS with highly different methylation levels between the two groups. To understand the role of these changes better, more thorough, single-cell RNA-seq and chromatin analyses should be performed. Most of the DMS were located in genes involved in functionally relevant neuronal pathways. However, while Riluzole seems to have a positive effect on dyskinesia in the rat LID model, its positive effect for this indication could not yet be demonstrated clinically. Therefore, by identifying potential candidate genes (such as *Reln*, *Dab1*, or glutamate receptor genes), our data may open new avenues for the study of the molecular mechanisms behind the development of LID. Furthermore, some of the genes identified here are potential therapeutic targets for LID treatment, either by conventional therapy (e.g., allosteric modulators of glutamate receptors) [7], or targeted DNA methylation modulation by epigenomic editing with the help of the Cas9-Tet1 system [77,78].

## Data Availability

GSE176004, https://www.ncbi.nlm.nih.gov/geo/guery/acc.cgi?acc=GSE176044 (accessed on 1 June 2021).

## Supplementary Materials

The following are available online at https://www.mdpi.com/article/10.3390/cells10061442/s1, Table S1 and Legend_Supplementary Table S1.
