# Supplementary material for: Riluzole Administration to Rats with Levodopa-Induced Dyskinesia Leads to Loss of DNA Methylation in Neuronal Genes
Source: Cells. 2021 Jun 9;10(6):1442. doi: 10.3390/cells10061442 (PMC8228416; doi:10.3390/cells10061442)
Supplement: Supplementary file 1 [file cells-10-01442-s001.zip › Legend_Supplementary table 1.pdf]

Legend

Supplementary table 1.

Table 1a. Indicates the results of the comparison of 3 control and 3 lesioned striata within the same treatment groups. Number of hypo- and hypermethylated DMS are shown separately for each comparison. Last column shows the sum of the DMS within each group. UNT: untreated (lesion only), L-DOPA: lesion + L-DOPA, L-DOPA+R: lesion + L-DOPA+Riluzole, c: control side, l: lesioned side

Table 1b. Indicates the results of the comparison of striata between different treatment groups. In each comparison 3-3 striata were compared, controls with controls and lesioned with lesioned. Number of hypo- and hypermethylated DMS are shown separately for each comparison. Last column shows the sum of the DMS within each group.

Table 1c. Indicates the results of the comparison of striata between different treatment groups. All 6 striata from a treatment group were compared to the 6 striata of the other.

Table 1d. DMS analysis of L-DOPA vs UNT groups. Column A: CpG ID (all analyzed CpGs were numbered), column B-E: chromosomal location, column F: significance, column G: corrected P value (q value), column H: methylation difference between the L-DOPA and UNT, column I: gene intronic region, column J: gene exonic region, column K: gene promoter gerion

Table 1e. DMS analysis of L-DOPA+R vs UNT groups.

Table 1f. DMS analysis of L-DOPA+R vs L_DOPA groups.
